# Association between zinc status and autism spectrum disorder in children and adolescents: a systematic review and meta-analysis of case–control studies

**DOI:** 10.3389/fnut.2025.1710999

**Published:** 2025-11-24

**Authors:** Hezuo Liu, Ji Chen, Jia He, Xuening Li

**Affiliations:** 1 Ninghai Maternal and Child Health Hospital, Ningbo, Zhejiang, China; 2 The Fourth Affiliated Hospital of China Medical University, Shenyang, Liaoning, China

**Keywords:** zinc deficiency, autism spectrum disorder, children, adolescents, meta-analysis

## Abstract

**Background:**

This study aimed to further corroborate a previously reported connection between zinc nutritional status and the occurrence of autism spectrum disorder (ASD) among children and adolescents.

**Methods:**

Following the Preferred Reporting Items for Systematic Reviews and Meta-Analyses (PRISMA) 2020 guidelines, a systematic review and meta-analysis was conducted. The PubMed, Embase, Web of Science, Scopus, and PsyclNFO databases were searched for all relevant case–control studies published until January 2024. Cohen’s kappa was computed to assess reviewer agreement. This meta-analysis used a random-effects model to summarize the overall association between zinc levels and ASD. The Q-test and I^2^ statistics were used to evaluate the heterogeneity of the studies, while funnel plots, Begg’s test, and Egger’s test were used to evaluate publication bias.

**Results:**

We included 25 case–control studies with 4,763 children and adolescents, comprising 2,499 cases and 2,264 typical controls. The random-effects meta-analysis revealed that whole blood and plasma/serum zinc levels were negatively associated with ASD (standardized mean difference [SMD] = −0.44, 95% confidence interval [CI]: −0.63 to −0.25; SMD = −1.79, 95% CI: −2.74 to −0.84), whereas hair (SMD = −0.01, 95% CI: −0.40 to 0.37) and urinary (SMD = −0.17, 95% CI: −0.87 to 0.53) zinc levels were not associated with ASD. Moreover, we observed statistically significant heterogeneity among the included studies (plasma/serum zinc: I^2^ = 98.8%, *P*<0.001; hair zinc: I^2^ = 88.4%, *P*<0.001; urinary zinc: I^2^ = 88.0%, *P*<0.001).

**Conclusion:**

Blood zinc levels were associated with ASD among children and adolescents. Therefore, screening blood zinc levels in children with ASD may be warranted. Further prospective studies are warranted to elucidate the role of zinc in the etiology of ASD.

## Introduction

Autism spectrum disorder (ASD) is a group of neurodevelopmental disorders characterized by impaired social interaction, repetitive stereotyped behaviors, and narrowed interests, which can lead to substantial disability across the life cycle (1). Recent research suggests that the global prevalence of ASD is 0.6%, with the prevalence in Asia, America, Europe, Africa, and Australia reported as 0.4, 1, 0.5, 1, and 1.7%, respectively (2). Although many studies have shown that genetic, environmental, and immunological factors play important roles in the etiology of ASD, the exact mechanisms remain unclear (3). Research suggests that nutrients, as an important environmental factor, may also be associated with ASD development (4).

Zinc is an essential trace element in the human body. It serves as a component and activator of metalloenzymes and is related to the activity of over 300 enzymes (5). Zinc deficiency can lead to reduced activity of zinc-containing enzymes, such as DNA and RNA polymerases, thereby decreasing the levels of DNA and RNA in nerve cells and affecting cell division and proliferation (e.g., in hippocampal progenitor cells). These effects can consequently influence the development of brain structure and function in children (6, 7). Therefore, zinc plays an important role in maintaining the normal structure and functions of the central nervous system, especially during the first 1,000 days of life (8). Severe zinc deficiency can lead to neuropsychological changes such as emotional instability, irritability, and depression (5, 9). Animal studies have shown that zinc deficiency, including during pregnancy, leads to ASD-related behaviors such as impaired social communication, communication disorders, and repetitive stereotyped behaviors (10, 11). In addition, eating-related problems, such as fussy eating, preference for specific foods, and stereotyped eating behavior, are more common among children with ASD than among those without ASD (12). Therefore, children with ASD may get even less zinc from food, potentially further aggravating ASD-related behaviors.

Epidemiological investigations have shown that serum zinc levels were significantly lower among children with ASD than among those without ASD (13, 14). Clinical trials have reported that zinc supplementation can alleviate clinical symptoms in children with zinc deficiency and ASD (15). Moreover, a clinical study found that zinc supplementation in children with ASD increased cognitive motor ability (16). However, some studies found that the zinc nutritional status was significantly higher among children with ASD than among children in the control group (17). Moreover, other studies did not find any association between zinc nutritional status and ASD (18, 19). Therefore, the association between zinc status and ASD remains inconsistent in population studies.

Although a systematic review of zinc status and ASD has recently been published (6), it only reported lower zinc concentrations among individuals with ASD and lacked a detailed statistical examination, such as a quantitative analysis of the combined effect size. In addition, given the rising prevalence of ASD and the importance of zinc for physical and mental development, further studies on the relationship between zinc nutritional status and ASD in children and adolescents are necessary to corroborate the previously established connection. We believe that our research will be of interest to some researchers. Therefore, we aimed to conduct a meta-analysis to review and summarize the available evidence from observational studies and clarify the association between zinc and ASD and to further provide evidence for zinc supplementation in children with ASD.

## Methods

### Search strategy

We conducted this study in accordance with the Preferred Reporting Items for Systematic Reviews and Meta-Analyses (PRISMA) guidelines (20). The study protocol was registered in the International Platform of Registered Systematic Review and Meta-analysis Protocols (INPLASY; registration number: INPLASY202450023). Electronic searches of the PubMed, Embase, Web of Science, Scopus, and PsyclNFO databases for English-language literature were conducted from their inception to 13 January 2024. In addition, we manually reviewed the list of references included in the articles to avoid the potential omission of relevant articles. The search included a combination of MeSH words and free text words as follows: “Trace Elements,” “Zinc,” “Zinc levels” or “Trace Element” and “Autism,” “Autism Spectrum Disorder,” “ASD,” or “Autistic Disorder.” Supplementary Table S1 presents the search strategy used for each database.

### Study selection

The inclusion criterion was case–control investigations involving children and adolescents aged 2–18 years. The diagnosis relied on either the Diagnostic and Statistical Manual of Mental Disorders (DSM) or the International Classification of Diseases-10 (ICD-10) (21). The zinc concentrations in eligible ASD children were analyzed by measuring zinc levels in biological specimens. All included studies were required to provide comprehensive data. Reviews, animal experiments, meeting minutes, redundant literature (such as same themes and same cohort), and studies involving additional psychiatric disorders were excluded.

### Data extraction and quality assessment

Reviewers independently and redundantly extracted the following details from the studies: surname of the primary author, year of publication, geographical setting of the study, age of the participants, sample size, sample source, analytical method used to detect zinc, and zinc levels (mean ± standard deviation) in both the case and control groups. Three investigators (Hezuo Liu, Ji Chen, and Jia He) independently extracted the literature data. Cohen’s kappa was computed to assess the inter-rater reliability during data extraction. The agreement between the two investigators was 95%. In case of disagreement, the third investigator (Xuening Li) decided to extract the literature data. If supplementary information was needed, direct communication via email was established with the principal authors.

The Newcastle–Ottawa scale (NOS) was used to evaluate each study using six items in three groups, including selection, exposure, and comparability. In addition to the *Comparability* item, each item could receive 1 point (1 star), with the total range being 0 to 2 stars (22). The final scores for the classification of the risk of bias were as follows: ≥7 stars, high quality; ≥4 and ≤6 stars, moderate quality; and <4 stars, low quality.

### Statistical analysis

Statistical analysis was conducted using Stata 12.0 software (Stata Corporation LLC, College Station, United States). Cohen’s kappa was computed to assess reviewer agreement. Standardized mean differences (SMDs) were pooled in the meta-analysis because the units were uniform across studies (23). Therefore, the association between zinc levels in whole blood, plasma/serum, hair, and urine with ASD was evaluated using a combination of SMDs and 95% confidence intervals (CIs). Heterogeneity among the studies was assessed using the I^2^ test, with I^2^ > 50% indicating substantial heterogeneity. Consequently, the random-effects model was used to merge the data due to high heterogeneity (24). Subgroup and sensitivity analyses were also performed to investigate the sources of heterogeneity. The subgroup analysis stratified the studies based on the sample source (serum, plasma, and whole blood), region of study (Europe and America, Russia, and Asia), year of study (≤2010 and >2010), zinc measurement method (inductively coupled plasma mass spectrometry [ICP-MS] and other methods), diagnostic criteria, and NOS score (≤6 and ≥7). Sensitivity analyses were performed using the metaninf test to assess the impact of each individual study on the overall result. Publication bias was assessed using funnel plots, Begg’s test, and Egger’s test. *p*-values of <0.05 were considered statistically significant, except for Begg’s test and Egger’s test, where the *p*-value was less than 0.1.

## Results

### Search results and study characteristics

**Figure 1:**
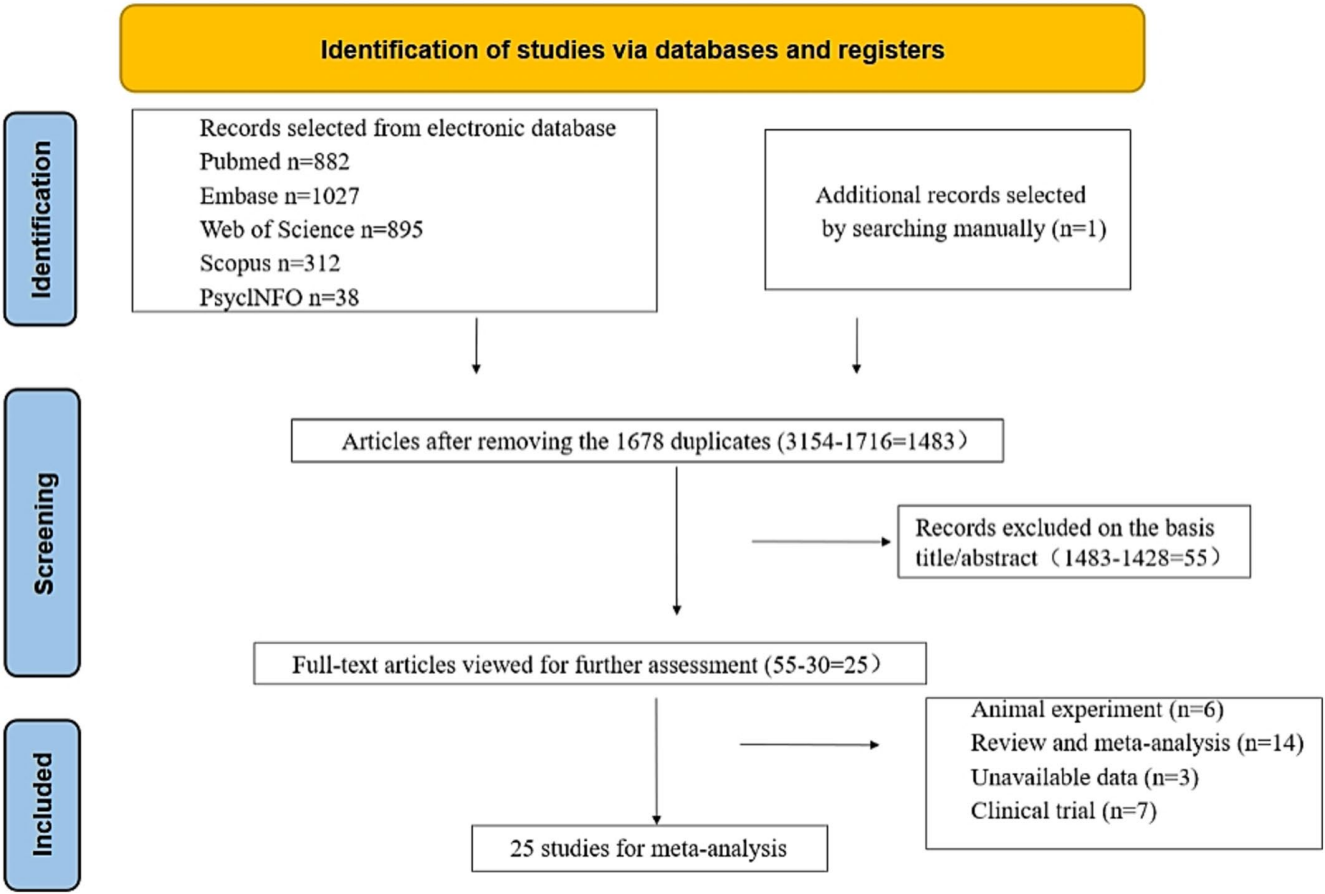
Flowchart of study selection.

We conducted a comprehensive search of the literature and examined 3,154 articles from various reputable databases, including PubMed, Embase, Web of Science, Scopus, and PsyclNFO. We identified one additional article by reviewing the reference lists of the relevant studies. We eliminated 1,716 duplicate articles through both automated and manual processes. Subsequently, we excluded 1,428 unrelated studies by carefully reviewing the titles and abstracts. Eventually, 55 studies were identified, which merited a thorough examination. After further scrutiny, 30 studies were deemed unsuitable for inclusion; the reasons for exclusion were 6 animal studies, 14 reviews or meta-analyses, 3 with unavailable data, and 7 clinical trials. Ultimately, our meta-analysis included 25 studies (Table 1) (13, 14, 17–19, 25–44). Among these, 14 studies involving 3,698 participants (case: 1,949 and control: 1,749) reported blood zinc levels, 11 studies involving 1,031 participants (case: 525 and control: 506) focused on hair zinc levels, and 3 studies involving 294 participants (case: 155 and control: 139) focused on urine zinc levels (Figure 1).

**Table 1:** Studies included in the meta-analysis.

Study	Country	Age (years)		Number of samples		Diagnostic criteria	Sample type	Zinc measurement method	NOS score
		Case	Control	Case	Control				
Rezaei et al. (25)	Iran	11.1 ± 2.26	10.4 ± 2.93	44	35	DSM-V	Urine	ICP-MS	8
Al-Farsiym et al. (26)	Oman	3–14	3–14	27	27	DSM-IV	Hair	ICP-MS	7
Russo et al. (27)	USA	11.7 ± 5.62	11.7 ± 5.62	79	18	DSM-IV	Plasma	ICP-MS	7
Zhao et al. (28)	China	4.2 ± 1.5	3.8 ± 1.3	30	30	ICD-10	Blood/Urine	ICP-MS	7
Qin (29)	China	4.2	4.3	34	38	DSM-IV	Blood	ICP-OES	7
Wu et al. (13)	China	4.94 ± 2.11	4.97 ± 2.66	113	141	DSM-V	Blood	FAAS	8
Graciun et al. (14)	Italy	5.83 ± 2.92	5.95 ± 2.92	28	28	DSM-IV	Blood	ICP-MS	8
Skalny et al. (18)	Russia	6.4 ± 2.9	6.3 ± 2.9	70	70	ICD-10	Hair/Serum	ICP-MS	8
Abdwahi et al. (30)	Malaysia	3–6	3–6	81	74	DSM-V	Urine	ICP-MS	7
Tinkov et al. (31)	Russia	2–7	2–7	30	30	ICD-10	Hair/Serum	ICP-MS	6
Wu et al. (32)	China	2–8	2–8	92	103	ICD-10	Serum	ICP-MS	7
Zhai et al. (17)	China	4.96 ± 1.01	4.90 ± 0.97	78	58	DSM-IV	Hair	ICP-MS	8
Skalny et al. (19)	Russia	5.12 ± 2.36	5.11 ± 2.34	74	74	Not reported	Hair	ICP-MS	6
Mehta et al. (33)	USA	2–4	2–4	52	22	Not reported	Serum	FAAS	7
Skalny et al. (34)	Russia	5.18 ± 1.0	5.13 ± 1.05	52	52	ICD-10	Hair	ICP-MS	8
Priya et al. (35)	India	4–12	4–12	45	50	ICD-10	Hair	AAS	6
Jackson (36)	English	7–16	7–17	12	30	Not reported	Plasma	NA	5
Li et al. (37)	China	3.78 ± 1.22	3.78 ± 1.22	60	60	DSM-IV	Serum	NA	6
Shearer et al. (38)	USA	8.4 ± 0.6	8 ± 0.8	12	12	Not reported	Hair	AAS	6
Adam et al. (39)	USA	3–15	3–15	51	40	Not reported	Hair	ICP-MS	7
Wecker (40)	USA	2–11	2–11	12	21	DSM	Hair	AAS	6
Guo et al. (41)	China	4.06 ± 1.13	4.24 ± 1.20	274	97	DSM-V	Serum	AAS	7
Sweetman et al. (42)	Ireland	2–16	2–16	74	72	DSM-V	Hair	ICP-MS	7
Zhang et al. (43)	China	4.3 ± 1.2	4.3 ± 1.3	1,020	1,038	ICD-10	Serum	ICP-MS	8
Adam et al. (44)	USA	5.16	5.16	55	44	Not reported	Plasma	ICP-MS	8

NA, not available; AAS, atomic absorption spectroscopy; DSM, Diagnostic and Statistical Manual of Mental Disorders; FAAS, flame atomic absorption spectroscopy; ICP-OES, inductively coupled plasma optical emission spectrometry; ICD-10, International Classification of Ddiseases-10; ICP-MS, inductively coupled plasma mass spectrometry; NOS, Newcastle–Ottawa scale.

Table 1 presents the primary features of the 25 case–control studies included in this meta-analysis, which were published between 1978 and 2023. Among the 14 studies (case: 1,949 and control: 1,749) examining the correlation between blood zinc and ASD, 7 focused on serum zinc (case: 1,598 and control: 1,420), 3 focused on plasma zinc (case: 146 and control: 92), and 4 focused on the zinc concentration in whole blood (case: 205 and control: 237). Furthermore, Table 2 provides the quality assessments of the included studies.

**Table 2:** Quality assessment of the included studies.

Study	Case definition adequate	Representativeness of the cases	Selection of controls	Definition of controls	Comparability of cases and controls	Ascertainment of exposure	Same method of ascertainment	Non-response rate	NOS score
Rezaei et al. (25)	*	*	*	—	**	*	*	*	8
Al-Farsiym et al. (26)	*	*	—	—	**	*	*	*	7
Russo et al. (27)	*	*	—	—	**	*	*	*	7
Zhao et al. (28)	*	*	—	—	**	*	*	*	7
Qin (29)	*	*	*	*	—	*	*	*	7
Wu et al. (13)	*	*	—	*	**	*	*	*	8
Graciun et al. (14)	*	*	—	*	**	*	*	*	8
Skalny et al. (18)	*	—	*	*	**	*	*	*	8
Abdwahi et al. (30)	*	*	*	*	—	*	*	*	7
Tinkov et al. (31)	*	*	—	*	**	—	*	*	6
Wu et al. (32)	*	*	*	*	—	*	*	*	7
Zhai et al. (17)	*	*	—	*	**	*	*	*	8
Skalny et al. (19)	—	*	—	*	**	*	*	*	6
Mehta et al. (33)	*	*	*	*	—	*	*	*	7
Skalny et al. (34)	*	*	—	*	**	*	*	*	8
Priya et al. (35)	*	*	—	*	**	*	*	*	6
Jackson (36)	*	—	*	*	—	—	*	*	5
Li et al. (37)	*	*	—	*	**	—	*	*	6
Shearer et al. (38)	—	*	*	*	—	*	*	*	6
Adam et al. (39)	—	*	—	*	**	*	*	*	7
Wecker (40)	*	*	—	*	—	*	*	*	6
Guo et al. (41)	*	*	*	*	—	*	*	*	7
Sweetman et al. (42)	*	*	*	*	—	*	*	*	7
Zhang et al. (43)	*	*	—	*	**	*	*	*	8
Adam et al. (44)	*	*	—	*	**	*	*	*	8

NOS, Newcastle–Ottawa scale.

### Overall meta-analysis

**Figure 2:**
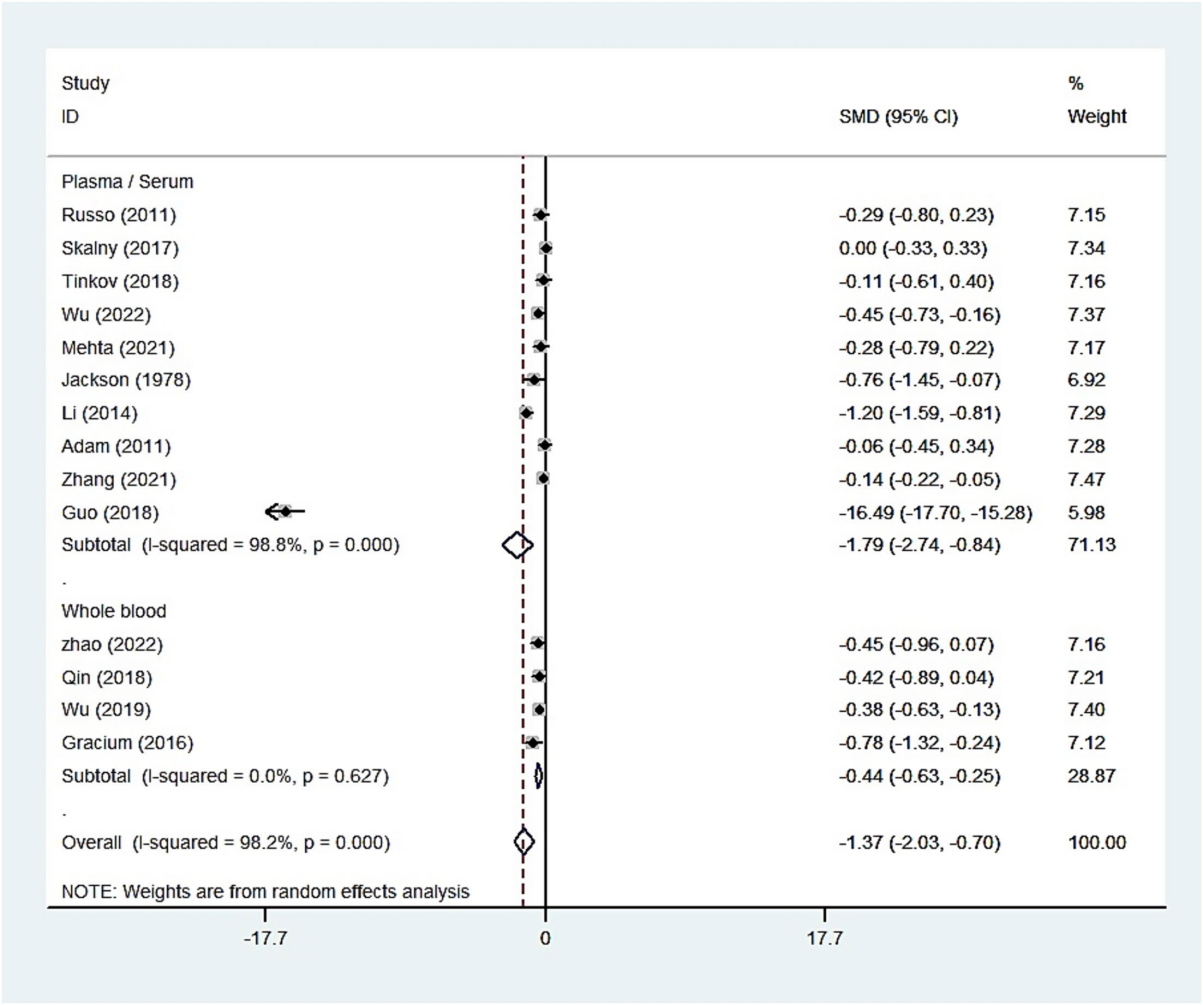
Forest plot of the blood zinc levels in children with autism spectrum disorder and controls.

**Figure 3:**
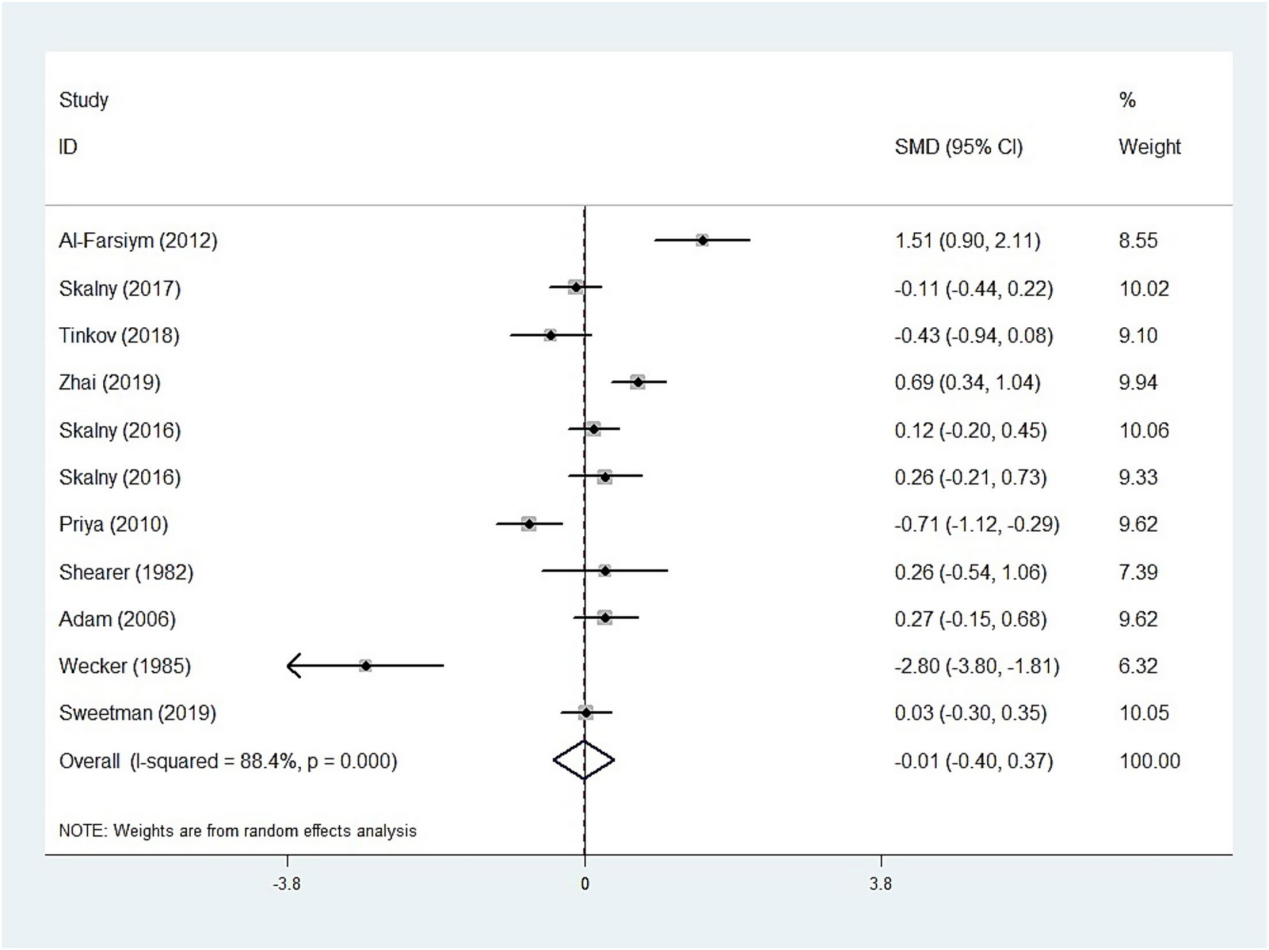
Forest plot of hair zinc levels in children with autism spectrum disorder and controls.

**Figure 4:**
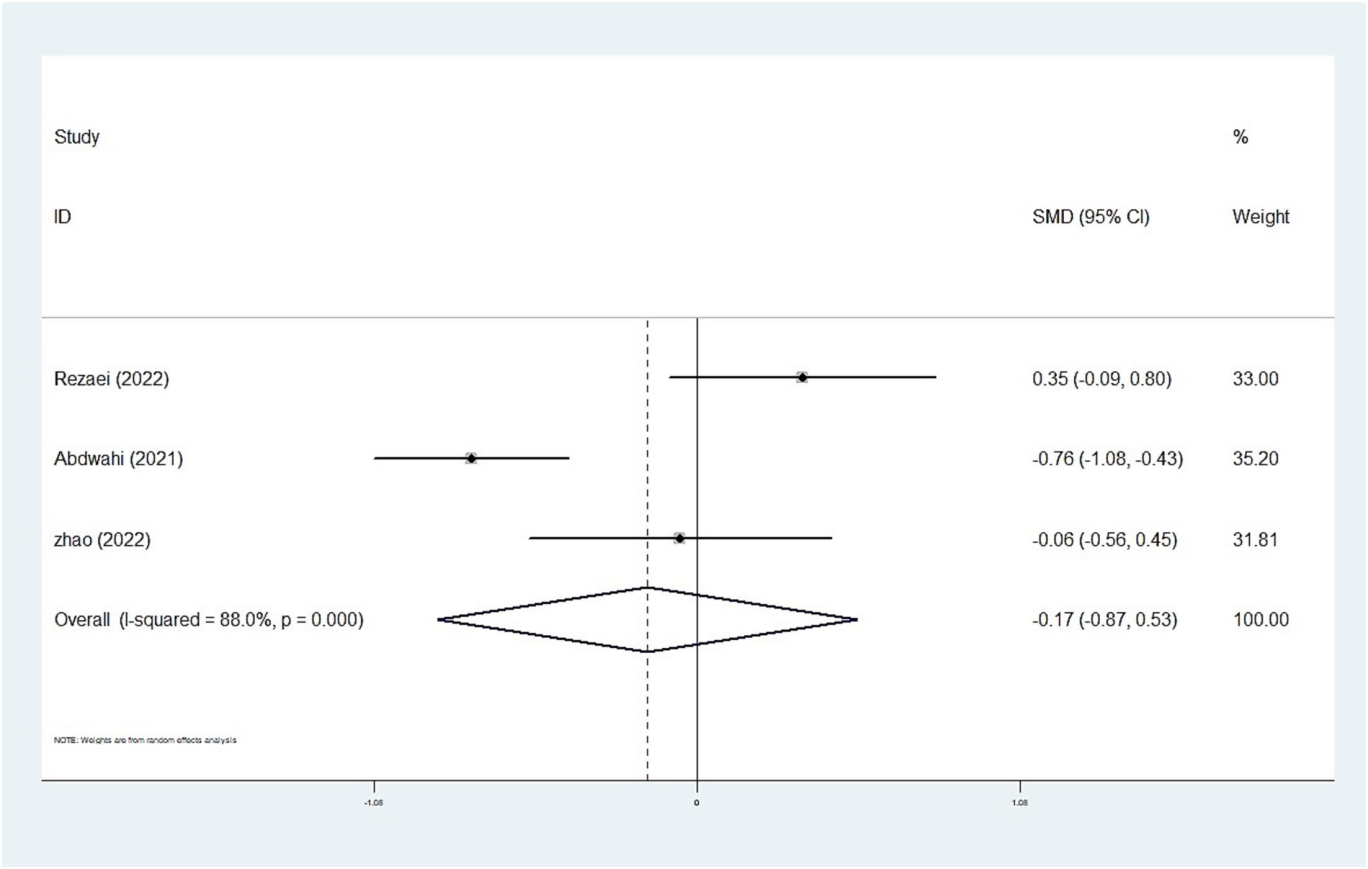
Forest plot of urine zinc levels in children with autism spectrum disorder and controls.

The whole blood and plasma/serum zinc levels were negatively associated with ASD (SMD = −0.44, 95% [CI]: −0.63 to −0.25; SMD = −1.79, 95% CI: −2.74 to −0.84), while hair (SMD = −0.01, 95% CI: −0.40 to 0.37) and urinary (SMD = −0.17, 95% CI: −0.87 to 0.53) zinc levels were not associated with ASD (Figures 2–4). Nevertheless, the included studies exhibited significant heterogeneity (plasma/serum zinc: I^2^ = 98.8%, *P*<0.001; hair zinc: I^2^ = 88.4%, *P*<0.001; urinary zinc: I^2^ = 88.0%, *P*<0.001).

### Subgroup analyses and sensitivity analyses

**Figure 5:**
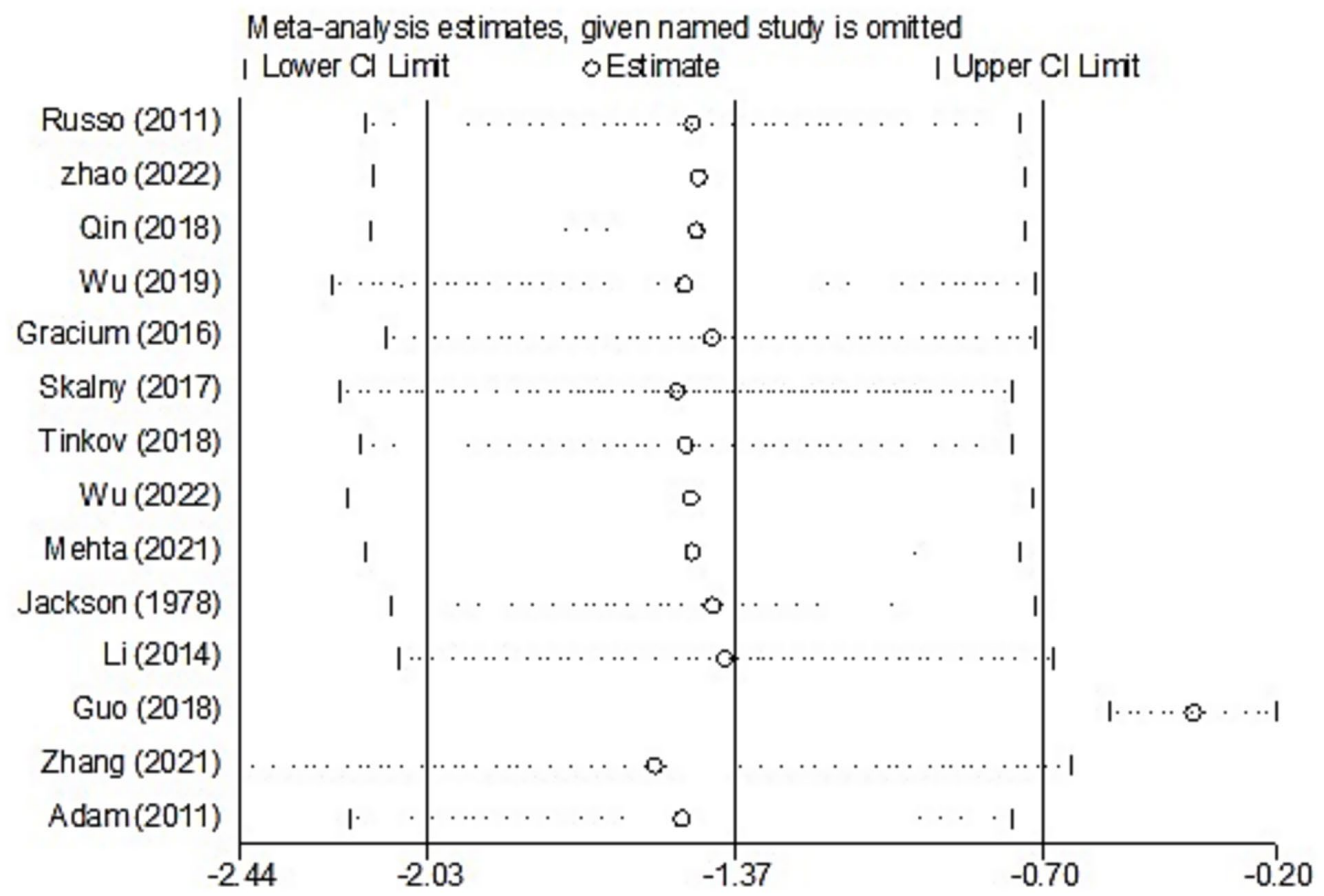
Leave-one-out sensitivity analysis (blood zinc).

**Figure 6:**
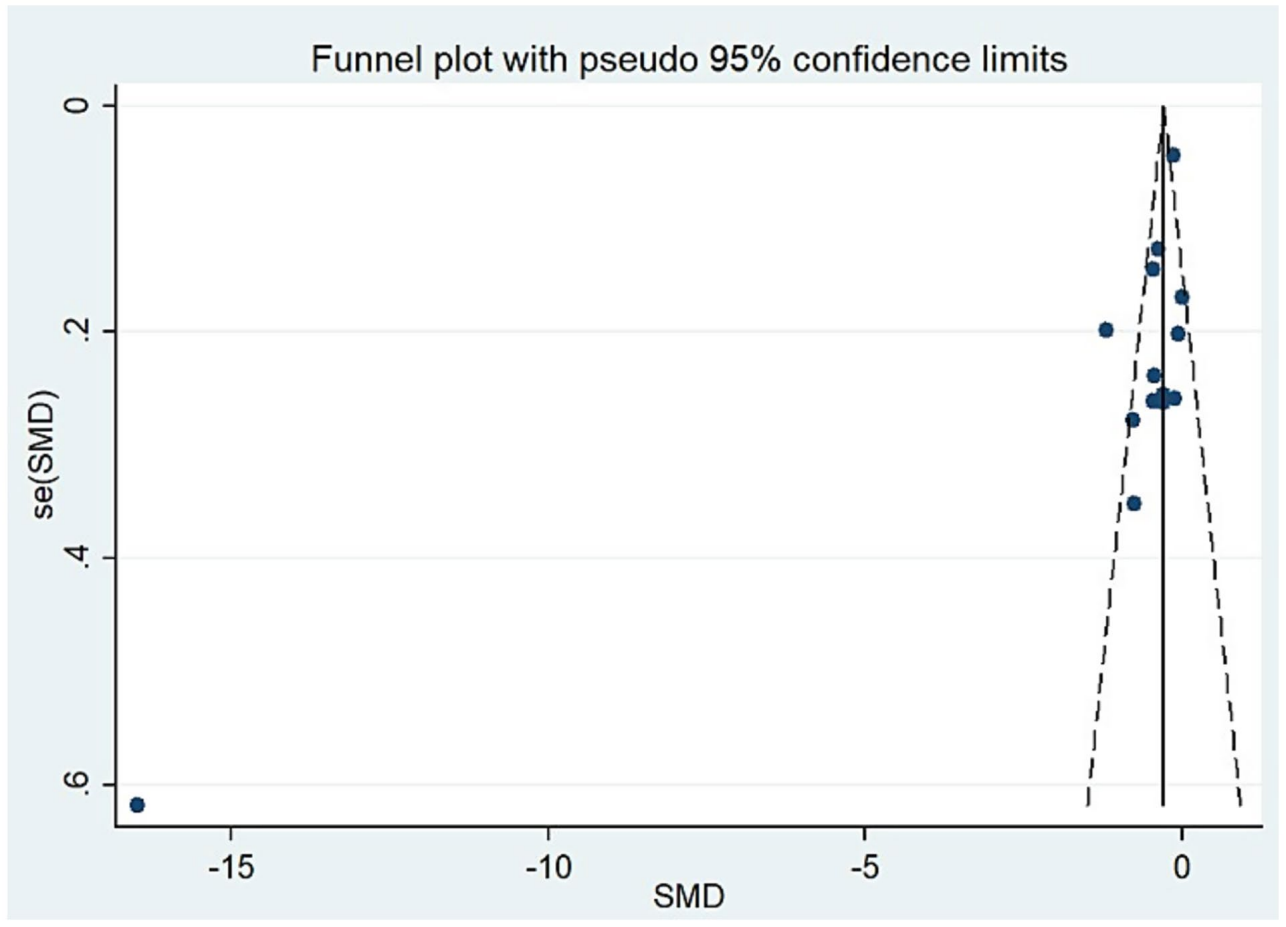
The funnel plot for the test of publication bias (blood zinc).

**Figure 7:**
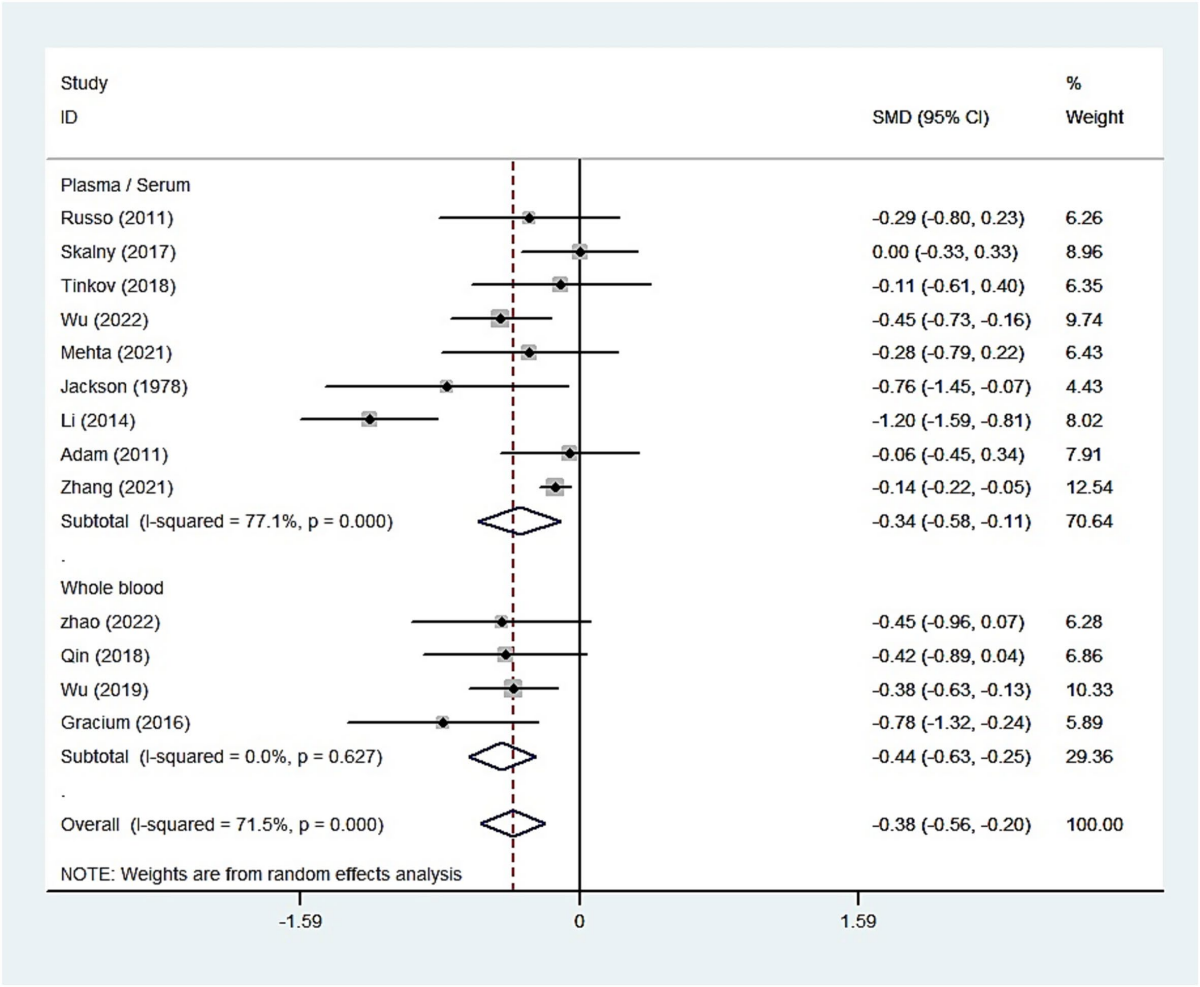
Forest plot of the blood zinc levels in children with autism spectrum disorder and controls [excluding Guo et al. (41)].

For studies analyzing blood zinc levels, a subgroup analysis was performed. The heterogeneity was reduced for the region of study (Russia), sample source (whole blood), and zinc assessment method (ICP-MS) (Table 3). Given the heterogeneity of our study, we conducted a sensitivity analysis. After removing each article individually, the sensitivity analysis indicated that the study by Guo et al. (41) was a clear outlier (Figure 5). In addition, the results indicated publication bias in the meta-analysis (Begg’s test, *p* = 0.063; Egger’s test, *p* = 0.099). The funnel plot was asymmetrical, due to the inclusion of the study (Figure 6), which altered the pooled estimate for plasma/serum (SMD = −0.34, 95% CI: −0.58 to −0.11) and reduced heterogeneity (I^2^ = 77.1%, *P*<0.001) (Table 3; Figure 7). However, no substantial modifications were observed (Table 3).

**Table 3:** Subgroup analysis to assess the blood and hair zinc levels in children with autism spectrum disorder.

Subgrouped by	No.	*SMD*	95% CI	*P-*significance test(s) of SMD	*P*-heterogeneity intergroup	I^2^ (%)
Studies on hair zinc						
Overall	11	−0.01	−0.40 to 0.37	0.058	<0.001	88.4
Region of study						
Europe and America	5	−0.07	−0.97 to 0.82	0.871	<0.001	92.6
Russia	4	−0.14	−0.38 to 0.09	0.231	0.193	36.5
Asia	2	−0.00	−1.37 to 1.37	0.998	<0.05	96.1
Zinc assessment method						
ICP-MS	8	0.19	−0.14 to 0.53	0.262	<0.001	83.5
AAS	3	−1.04	−2.43 to 0.35	0.142	<0.001	91.1
Year						
≤2010	5	−0.49	−1.20 to 0.22	0.173	<0.001	90.0
>2010	6	0.22	−0.25 to 0.69	0.358	<0.001	88.0
NOS score						
≤6	5	−0.19	−0.85 to 0.46	0.562	<0.001	91.5
≥7	6	0.07	−0.35 to 0.49	0.733	0.001	82.8
Studies on blood zinc						
Overall	14	−1.37	−2.02 to −0.70	<0.001	<0.001	98.2
Overall [excluding Guo et al. (41)]	13	−0.38	−0.56 to −0.20	<0.001	<0.001	71.5
Region of study						
Europe and America	7	−0.54	−0.85 to −0.23	<0.001	0.003	70.1
Russia	2	−0.03	−0.31 to 0.25	0.824	0.734	0.0
Asia	5	−3.37	−5.16 to −1.58	<0.001	<0.001	99.4
Asia [excluding Guo et al. (41)]	4	−0.26	−0.44 to −0.09	<0.001	0.153	43.1
Zinc assessment method						
ICP-MS	8	−0.23	−0.38 to −0.08	0.003	0.111	40.2
Others	6	−3.16	−5.32 to −1.00	0.004	<0.001	99.2
Others [excluding Guo et al. (41)]	5	−0.60	−0.96 to −0.24	0.001	0.007	71.8
NOS score						
≥7	11	−1.75	−2.62 to −0.88	<0.001	<0.001	98.7
≥7 [excluding Guo et al. (41)]	10	−0.32	−0.46 to −0.18	<0.001	0.307	15.2
≤6	3	−0.53	−1.14 to 0.09	0.092	<0.001	84.9
Sample source						
Whole blood	4	−0.44	−0.63 to −0.25	<0.001	0.627	0.0
Plasma	3	−0.28	−0.65 to 0.08	0.125	0.223	33.3
Serum	7	−2.46	−3.74 to −1.17	<0.001	<0.001	99.2
Plasma/serum	10	−1.79	−2.74 to −0.84	<0.001	<0.001	98.8
Serum [excluding Guo et al. (41)]	6	−0.36	−0.66 to −0.05	0.022	<0.001	84.3
Plasma/serum (excluding Guo et al.)	9	−0.34	−0.58 to −0.11	0.005	<0.001	77.1

The study by Guo et al. (41) was excluded because it was a clear outlier; SMD, standardized mean difference; CI, confidence interval; AAS, atomic absorption spectroscopy; ICP-MS, inductively coupled plasma mass spectrometry; NOS, Newcastle–Ottawa scale.

**Figure 8:**
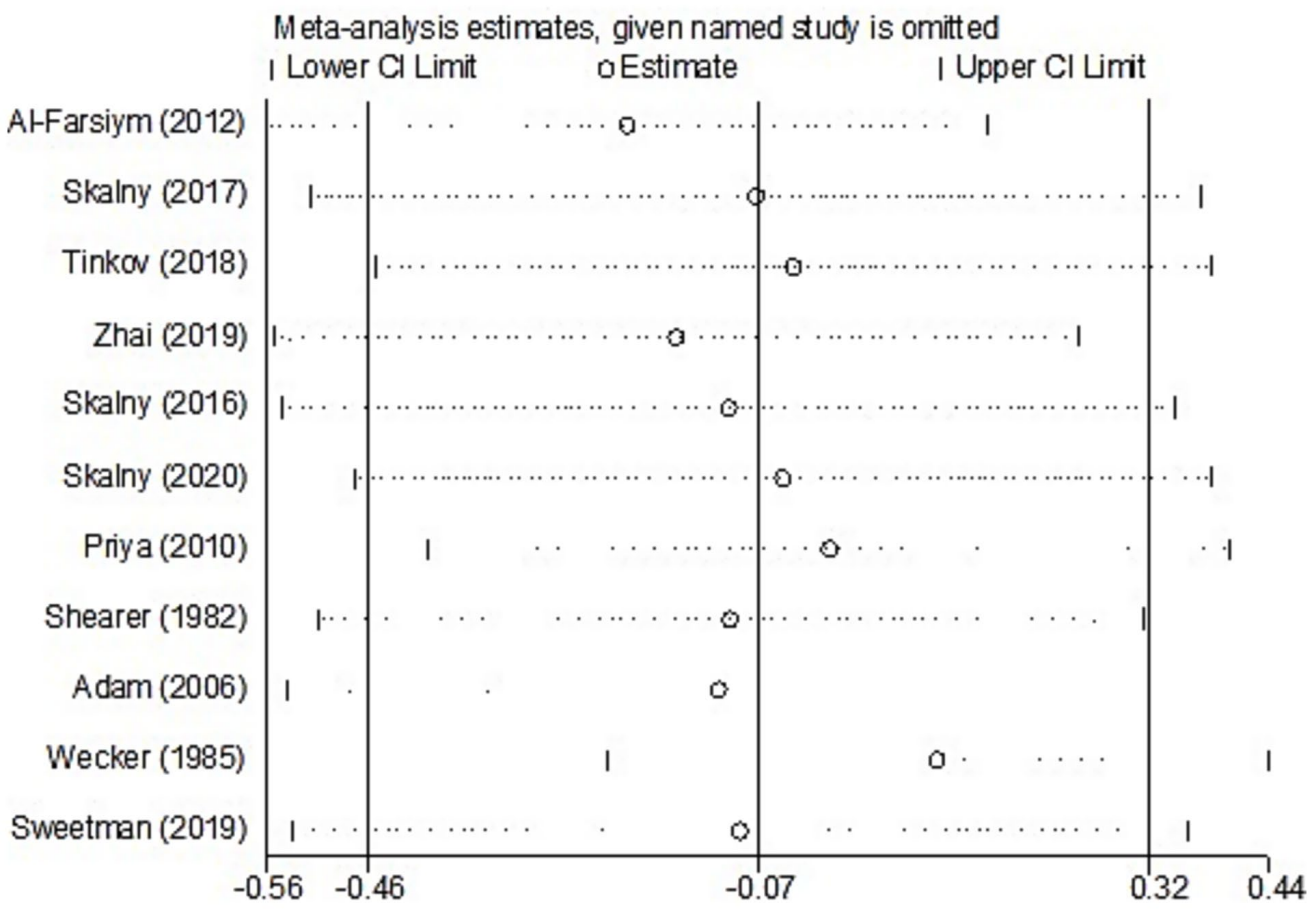
Leave-one-out sensitivity analysis (hair zinc).

**Figure 9:**
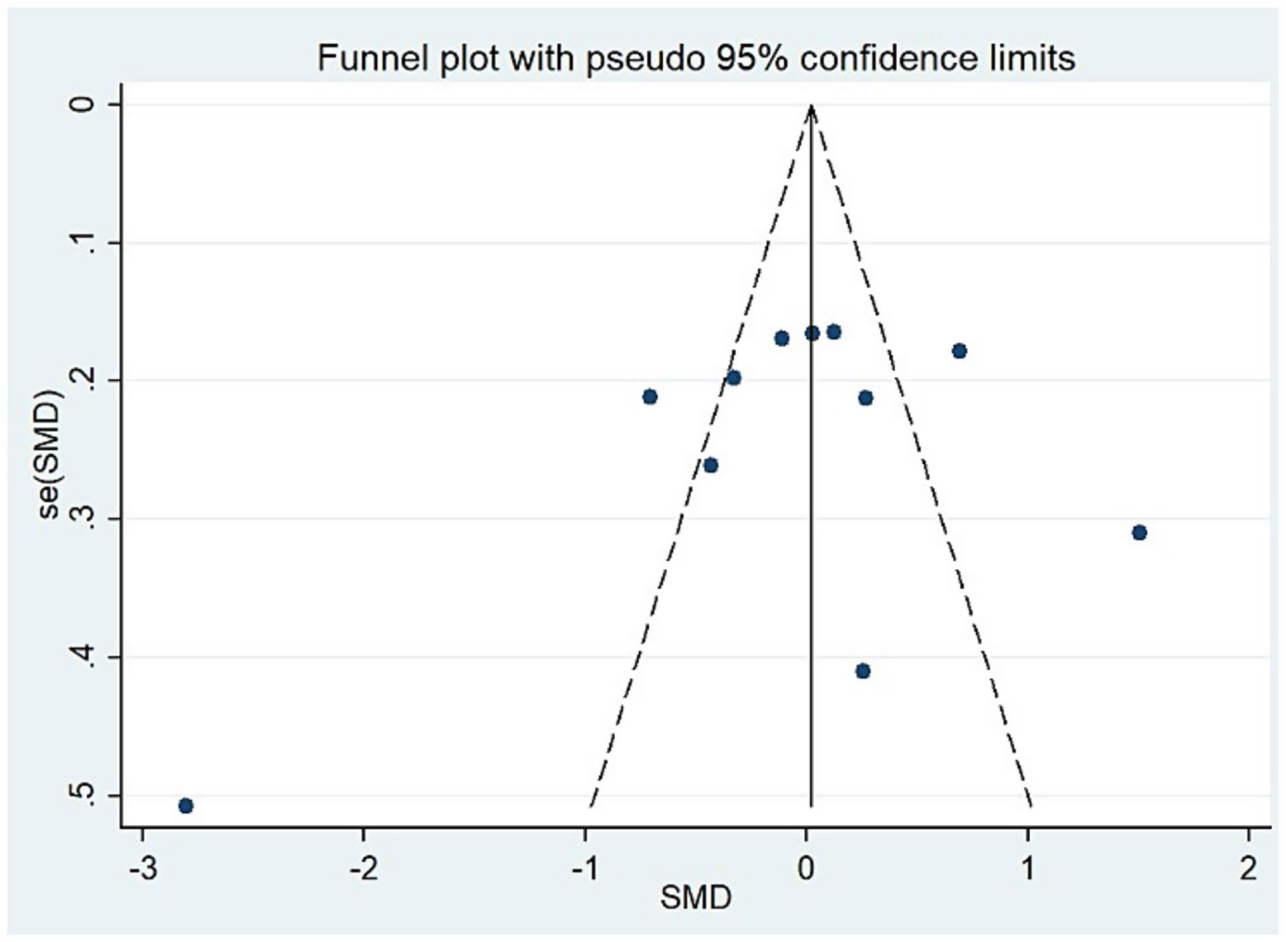
The funnel plot for the test of publication bias (hair zinc).

For studies analyzing hair zinc levels, subgroup analysis was performed based on the region of study (Europe and America, Russia, and Asia), year of study (≤2010 and >2010), zinc assessment method (ICP-MS and other methods), and NOS score (≤6 and ≥7). The heterogeneity was reduced for the region of study (Russia) (Table 3). After removing each article individually, the sensitivity analysis demonstrated that the combined results remained unchanged (Figure 8). The observations did not indicate any publication bias in the meta-analysis (Begg’s test, *p* = 0.484; Egger’s test, *p* = 0.450) (Figure 9).

## Discussion

The meta-analysis included 25 studies involving 4,763 participants. The results suggested a statistically significant difference in blood zinc levels between children with ASD and those in the control group, whereas no statistical significance was observed in urine or hair zinc levels. Substantial heterogeneity was present among the included studies. This meta-analysis found that children with ASD have lower blood zinc levels than children in the control group. Another meta-analysis found an association between plasma zinc and ASD, but no subgroup analysis was conducted (45). The majority of studies on zinc and ASD discussed in this meta-analysis were published before 2012; however, subsequent studies examining the correlation between zinc and ASD have been conducted since 2011. Our comprehensive meta-analysis included 25 surveys, 17 of which were published after 2011. Therefore, it was imperative to delve further into the relationship between zinc and ASD.

There was substantial heterogeneity among the included studies. The high heterogeneity observed in this meta-analysis may be attributed to various variables, such as demographics, sample source, assessment method, and adjusted statistical parameters. Therefore, the random-effects model was used to pool the data, and subgroup and sensitivity analyses were performed to investigate the sources of heterogeneity (24). The heterogeneity was reduced for the sample source and the zinc assessment method when exploring the sources of heterogeneity in studies on blood zinc. In our analysis, blood zinc levels included whole blood, serum, and plasma, which also accounted for the high heterogeneity observed. Previous studies have suggested that zinc concentrations in platelets, polymorphonuclear cells, mononuclear cells, and erythrocytes are not effective biomarkers of zinc status (9). Furthermore, serum and plasma zinc concentrations do not differ significantly in their relation to zinc nutritional status and are therefore used interchangeably in systematic reviews. Therefore, in addition to conducting a separate analysis of whole blood, we also pooled data from plasma and serum studies. The results from these analyses similarly indicated an association between zinc and ASD. In addition, previous research has compared serum and plasma zinc measurements using the atomic absorption spectrometer (AAS), inductively coupled plasma optical emission spectrometer (ICP-OES), and ICP-MS. Despite yielding similar zinc concentrations, accuracy, and precision when standardized materials and methods are applied, serum and plasma zinc measurements consistently show the highest coefficients of variation—regardless of the analytical platform used (AAS, ICP-OES, or ICP-MS) (46). Therefore, future studies should focus on methodological improvements, particularly for ICP-MS, to improve measurement precision. We found that, in the majority of studies, in addition to measuring blood zinc levels, hair zinc measurements are also commonly used in research involving individuals with ASD due to their advantages as a relevant, non-invasive, and easily collectible method (6). However, hair metal measurements can be affected by external contamination that is not easily removed through simple washing; therefore, hair zinc level testing is generally not recommended. In addition, sensitivity analyses identified the study by Guo et al. (41) as a clear outlier. After removing this study, the combined outcome was slightly altered, and heterogeneity was reduced; however, no substantial changes were observed, and the conclusion that blood zinc levels are negatively associated with ASD remained unchanged.

There are many causes of zinc deficiency among children with ASD. In addition to the physiological factors associated with ASD, specific dietary behaviors, such as rigid and repetitive eating patterns, fussy eating, and refusal to try new foods, can directly affect zinc intake (12). Gastrointestinal changes in children with ASD are another important factor that can affect blood zinc levels (47). A total of eight out of ten children with ASD experience gastrointestinal disturbances or dysfunctions, with the most common symptoms being vomiting, diarrhea, nausea, gastroesophageal reflux, and abdominal pain or distension (6, 47). Gastrointestinal disturbances or dysfunctions can directly and significantly affect zinc status, as zinc is involved in mechanisms that maintain gastrointestinal homeostasis (6). In addition, other factors influencing zinc levels in children’s blood and hair, such as variations in other metals (e.g., copper, lead, and cadmium), should be considered (6, 48). Evidence suggests that low zinc concentrations in children with severe ASD are associated with high concentrations of toxic metals, including lead, mercury, barium, and lithium (6, 49). Zinc is related to metallothionein, which primarily regulates zinc homeostasis within brain cells. Metallothionein also has a detoxification effect when combined with other metals, such as lead, cadmium, and copper (50). Therefore, high levels of toxic metals and trace element deficiencies in children have negative effects on neurodevelopment (51).

Regarding the underlying mechanisms of zinc, recent studies suggest that its interaction with synaptic dysfunction plays an important role in the pathogenesis of ASD (5). Several studies have shown that zinc deficiency damages the synaptic proline-rich synapse-associated protein 2 (ProSAP)/Shank scaffold, leading to alterations in synapse formation, maturation, and plasticity, and is associated with ASD behavior (11, 52). N-methyl-D-aspartate receptor (NMDAR) function is highly modulated by zinc, which is co-released with glutamate and concentrated in postsynaptic spines (53). Zinc mobilization increased NMDAR signaling and significantly improved social interaction in a murine model of ASD lacking Shank2 (54). In hippocampal cell cultures, an ASD-like biometal profile leads to a reduction of NMDAR (NR/Grin/GluN) subunits 1 and 2a, as well as Shank gene expression, along with a reduction of synapse density, and zinc supplementation was shown to rescue the aforementioned alterations (55). Some studies have also suggested that other zinc-dependent signaling mechanisms may be involved in the pathogenesis of ASD, such as cytoskeleton-regulating cortactin binding protein 2, P2X7R-mediated signaling, and the gut–brain axis (5).

Generally, the most recent research data demonstrate the critical role of zinc metabolism in the pathogenesis of ASD. Meanwhile, our research also confirms the existence of an association between blood zinc and ASD among children and adolescents. However, there is a lack of information on the potential translation of zinc-targeted therapy in humans, especially studies exploring how zinc supplementation affects human neuronal or synaptic function (53). Animal studies have shown that SH3 and multiple ankyrin repeat domains proteins (SHANKs) and NMDARs are key targets for effective zinc-induced therapy. Therefore, zinc supplementation also needs to be specifically examined in ASD patients involving mutations in SHANKs and NMDARs (53).

This meta-analysis has some limitations. First, as the included studies did not account for gender differences, we cannot determine whether gender influences the observed association. In addition, ASD diagnosis is much more complicated in children under the age of 3 years. However, we were not able to stratify by age groups due to the wide age range in the included studies. Second, the studies analyzed in this article were case–control in design, making it difficult to completely avoid recall bias and preventing any inference of a causal relationship between zinc and ASD. Since the effect of zinc deficiency on ASD is more pronounced in early life (5), including during pregnancy, we attempted to collect cohort studies to examine this relationship; however, only one relevant article was found (56). Therefore, further prospective studies are warranted to elucidate the role of zinc in the etiology of ASD, although the study design is difficult. Given the absence of validated early-life biomarkers, participant ascertainment was necessarily restricted to individuals aged at least 2 years. This limitation precludes direct insights into the prenatal period, which animal model studies implicate as a critical window for initial zinc dysregulation during fetal neurodevelopment (10). Consequently, our assessment of older children—whose zinc status is subject to substantial modulation by postnatal dietary and lifestyle factors—likely captures a confounded and heterogeneous physiological state. This temporal misalignment is a plausible source of the inconsistencies observed in the broader epidemiological literature. The robust group differences measured despite this significant dilution bias underscore the strength and biological significance of the underlying association. Third, due to the considerable time span of the included studies, the diagnostic criteria for ASD varied (for instance, regarding the inclusion of Asperger’s syndrome), which could potentially influence our conclusions. Therefore, we conducted a sensitivity analysis with stratification based on diagnostic criteria (DSM, DSM-IV, DSM-V, and ICD-10). The results of this sensitivity analysis for blood zinc remained consistent and did not alter our overall conclusions (Supplementary Table S2).

## Conclusion

Our meta-analysis concluded that children and adolescents with ASD have lower blood zinc levels than their typical counterparts and that ASD is associated with zinc nutritional status. Therefore, screening blood zinc levels in children with ASD may be reasonable. Further prospective studies are warranted to elucidate the role of zinc in the etiology of ASD. In addition, in the absence of validated biomarkers for the early diagnosis of ASD, identifying reliable biomarkers remains a priority for future research.

## Data Availability

The datasets presented in this study can be found in online repositories. The names of the repository/repositories and accession number(s) can be found in the article/Supplementary material.
