# Supplementary material for: Association between zinc status and autism spectrum disorder in children and adolescents: a systematic review and meta-analysis of case–control studies
Source: Front Nutr. 2025 Nov 24;12:1710999. doi: 10.3389/fnut.2025.1710999 (PMC12682681; doi:10.3389/fnut.2025.1710999)
Supplement: Supplementary file 1 [file Table_1.docx]

**Supplementary Table S1**. Database Search Strategies

| Database | Search query |
| --- | --- |
| PubMed | (("Autism Spectrum Disorder "[Title/Abstract]) OR ("ASD "[Title/Abstract])) OR ("Autistic Disorder "[Title/Abstract])) OR ("Autism "[Title/Abstract])) OR ("ASD "[MeSH Terms])) AND (("Zinc levels " [Title/Abstract]) OR"Trace Element " [Title/Abstract]) OR ("Zinc "[MeSH Terms]))AND (("Child " [Title/Abstract]) OR"Childrent " [Title/Abstract]) OR"Adolescence " [Title/Abstract]) OR ("Child "[MeSH Terms])OR ("Adolescence "[MeSH Terms])) |
| Scopus | TITLE-ABS-KEY(zinc OR “Trace Element” OR “Zinc levels” ) AND TITLE-ABS-KEY(“Autistic Disorder” OR “Autism Spectrum Disorder” OR “Autism” OR “ASD” ) AND TITLE-ABS-KEY( “Child ”OR “Children ”OR ”adolescent ”) |
| Web of Science | ('Autistic Disorder'/exp OR 'ASD'/exp OR 'Autism Spectrum Disorder'/exp OR 'Autism'/exp) AND ('zinc'/exp OR 'Zinc levels'/exp OR 'Trace Element'/exp) AND ('Child'/exp OR 'Children'/exp OR 'adolescent'/exp) |
| Embase | ("Autistic Disorder"/exp OR "ASD"/exp OR "Autism Spectrum Disorder"/exp OR "Autism"/exp) AND ("zinc"/exp OR "Zinc levels"/exp OR "Trace Element"/exp) AND ("Child"/exp OR "Children"/exp OR "adolescent"/exp) AND [embase]/lim |
| PsyclNFO | (zinc OR “Trace Element” OR “Zinc levels” ) AND (“Autistic Disorder” OR “Autism Spectrum Disorder” OR “Autism” OR “ASD” ) AND ( “Child ”OR “Children ”OR ”adolescent ”) |
