# Supplementary material for: Association between zinc status and autism spectrum disorder in children and adolescents: a systematic review and meta-analysis of case–control studies
Source: Front Nutr. 2025 Nov 24;12:1710999. doi: 10.3389/fnut.2025.1710999 (PMC12682681; doi:10.3389/fnut.2025.1710999)
Supplement: Supplementary file 2 [file Table_2.docx]

| Subgrouped by | No. | *SMD* | 95% CI | *P-*Significance test(s) of SMD | *P*-Heterogeneity  intergroup | I^2^ (%) |
| --- | --- | --- | --- | --- | --- | --- |
| **Studies on hair zinc** |  |  |  |  |  |  |
| Overall | 11 | -0.01 | -0.40 to 0.37 | 0.058 | <0.001 | 88.4 |
| Diagnostic criteria |  |  |  |  |  |  |
| DSM | 1 | -0.28 | −3.80 to -1.81 | <0.001 | - | - |
| DSM-IV | 2 | 1.06 | 0.27 to 1.85 | 0.009 | 0.023 | 80.7 |
| DSM-V | 1 | 0.03 | −0.30 to 0.35 | 0.874 | - | - |
| ICD-10 | 4 | -0.37 | -0.63 to -0.11 | 0.005 | 0.177 | 39.1 |
| Not reported | 3 | 0.19 | -0.06 to 0.43 | 0.135 | 0.853 | 0 |
| **Studies on blood zinc** |  |  |  |  |  |  |
| Overall | 14 | -1.37 | -2.02 to -0.70 | <0.001 | <0.001 | 98.2 |
| Overall (exclude Guo et al) | 13 | -0.38 | -0.56 to -0.20 | <0.001 | <0.001 | 71.5 |
| Diagnostic criteria |  |  |  |  |  |  |
| DSM | 0 |  |  |  |  |  |
| DSM-IV | 4 | -0.69 | -1.12 to -0.25 | 0.002 | 0.018 | 70.2 |
| DSM-V | 2 | -8.42 | -24.21 to 7.37 | 0.296 | <0.001 | 99.8 |
| DSM-V (exclude Guo et al) | 1 | -0.20 | -0.36 to 0.13 | 0.003 | - | - |
| ICD-10 | 5 | −0.20 | −0.36 to −0.04 | 0.013 | 0.178 | 36.5 |
| Not reported | 3 | −0.28 | −0.64 to 0.08 | 0.123 | 0.223 | 33.36 |

Supplementary Table S2. Subgroup analysis to assess the blood and hair zinc levels in children with autism spectrum disorder
